# Re-exploring the value of surveillance cultures in predicting pathogens of late onset neonatal sepsis in a tertiary care hospital in southern Sri Lanka

**DOI:** 10.1186/s13104-018-3448-9

**Published:** 2018-05-29

**Authors:** Nayani Prasangika Weerasinghe, Dhammika Vidanagama, Bilesha Perera, Herath Mudiyanselage Meththananda Herath, Ajith De Silva Nagahawatte

**Affiliations:** 1 Department of Microbiology, Faculty of Medicine, University of Ruhuna, Inland Hill Road, PO Box 70, Galle, Sri Lanka; 2 Department of Microbiology, Teaching Hospital, Karapitiya, PO Box 70, Galle, Sri Lanka; 3 Department of Community Medicine, Faculty of Medicine, University of Ruhuna, PO Box 70, Galle, Sri Lanka; 4 Department of Medicine, Faculty of Medicine, University of Ruhuna, PO Box 70, Galle, Sri Lanka

**Keywords:** Empirical antibiotics, Neonatal infections, Surveillance swabs

## Abstract

**Objective:**

To identify the validity of surveillance cultures in predicting causative organism(s) of late onset neonatal sepsis.

**Results:**

Prospective analytical study was conducted from January to April 2011 at the Neonatal Intensive Care Unit, Teaching Hospital, Karapitiya, Galle, Sri Lanka. Fifty neonates were screened on admission and weekly thereafter for colonization with potential pathogens. On suspicion of infection, relevant samples were cultured and tested for antibiotic sensitivity. There were 55 episodes of clinically suspected infections including 33 nosocomial infections. One-third (17/55) of all clinically suspected infections were culture positive. Out of 55, only 33 episodes were clinically suspected nosocomial infections. Clinically suspected nosocomial infection rate was 50/1000 patient-days. Culture proven nosocomial infection rate was 13.61/1000 patient-days. Coliforms were the commonest clinical isolate (76%) and 2/3 of them produced extended spectrum β lactamase. More than 80% of the isolates causing late onset sepsis were sensitive to carbapenems and aminoglycosides. Sensitivity, specificity, positive predictive value and negative predictive value of surveillance cultures were 77.8, 37.5, 31.8 and 81.8%, respectively. Surveillance samples can be used to predict pathogens of late-onset sepsis. Broad-spectrum antibiotics (carbapenems, aminoglycosides) are recommended as empirical therapy for late-onset neonatal sepsis.

## Introduction

Neonatal infections continue to be a major cause of morbidity and mortality in newborns throughout the world [1, 2]. The increasing populations of very low birth weight (VLBW) premature infants, who now survive due to improved neonatal care, represent the group at highest risk for infections. Neonatal infections are divided into two main categories, early onset sepsis (EOS); infections occurring during the first 48 h of life and late onset sepsis (LOS); infections occurring thereafter.

Neonatal sepsis causes approximately 1.6 million neonatal deaths annually in developing countries [3]. A Malaysian study reports rates of neonatal sepsis of 5–10% with case fatality rates 23–52% [4]. Klein et al. have shown that neurological sequelae as a complication in 20–30% of survivors of neonatal bacteraemia and 40% or more of those with meningitis [5].

NICU at the Teaching Hospital Karapitiya (THK) is among the few NICUs with level 3 facilities in the country. It receives neonates with various surgical and medical problems from other institutions in Southern province of Sri Lanka. Hospital data from the Microbiology and Infection Control Unit of THK showed that during 2009 and 2010 there had been three clusters of neonatal sepsis caused by extended spectrum beta lactamase (ESBL) producing coliforms in this NICU with increased mortality. Delayed first effective antibiotic dose due to inappropriate empirical antibiotic therapy is a main reason for delayed recovery and high mortality [6, 7]. It is noted that prospective studies on neonatal infections and surveillance cultures have not been conducted in this unit previously making it is a timely necessity to identify the causative pathogens and their current susceptibility patterns. The main objective of this study was to examine the validity of surveillance cultures in predicting causative organism(s) of late onset neonatal sepsis.

## Main text

### Materials and methods

A hospital based prospective study was conducted with a cohort of fifty neonates admitted to the NICU at the THK from 1st January 2011 to 30^th^ April 2011. Babies whose age was less than 28 days on admission to the NICU were recruited for the study and they were followed until 48 h of discharge from the NICU or for 48 h after completing 28 days of age while in the NICU. When assessing the value of surveillance cultures in predicting pathogens of LOS, only the episodes of infections developed following 48 h of admission to the NICU, were considered. The infection episodes present on admission to the unit were excluded from the analysis. However, if they developed another episode of infection during the NICU stay those occasions were considered for the analysis. Therefore only 33 episodes of LOS acquired during the NICU stay among 50 neonates were analyzed to assess the value of surveillance cultures. Data regarding patients’ demography, risk factors for sepsis, signs and symptoms, investigation results and antibiotic treatment were recorded. A peripheral blood culture, deep ear swab, nasal swab, umbilical swab and a rectal swab were collected for culture, from every neonate on admission to the unit. During the stay, babies were screened weekly with a rectal swab and respiratory tract secretion cultures; pharyngeal aspirates (PA) and gastric aspirates (GA) from non-intubated babies and endotracheal (ET) secretions from intubated babies. Clinical suspicion of sepsis in babies was based on criteria from different studies [3] and decision of the clinicians treating patients. Haematological and biochemical parameters were also used to support the diagnosis of sepsis. Appropriate clinical specimens were collected from babies for culture. Centre for Disease Control and Prevention (CDC) guidelines were followed in collection and transport of specimens [8]. Prepared guidelines were given to staff of the NICU for proper specimen collection and transport for bacteriological cultures. Specimens were cultured and organisms were identified according to standard operating procedures given by Sri Lanka College of Microbiologists in Laboratory Manual in Microbiology, which is again based on CDC guidelines [9]. Organisms were identified using colony morphology, microscopic features and routine bio-chemical tests like catalase test, coagulase test and oxidase test. API 20E and streptococcal grouping test kits were used to identify blood culture isolates of Enterobacteriaceae and streptococci. Antibiotic sensitivity testing (ABST) and interpretation of results were done according to protocols given by Clinical Laboratory Standards Institute [10]. ABSTs were performed by disc diffusion method or E strip method, on all significant isolates. The culture and ABST results of surveillance and clinical samples were documented. Most effective empirical antibiotic therapy was decided using the ABST patterns of clinical isolates.

Descriptive analysis of the sample subjects in relation to age, gestational age at birth, birth weight, EOS and LOS were conducted. Rates of EOS and LOS were calculated per 1000 admissions. Rate of nosocomial infections were calculated for 1000 patient-days of observation. Antibiotic resistance rates were calculated as percentages.

Sensitivity, specificity, positive predictive value and negative predictive value of surveillance cultures in predicting organisms causing subsequent infections were calculated.

### Results

Admission age ranged from 1 to 27 days and male babies consisted 58% of the sample. Birth weights ranged from 0.7 kg to 3.6 kg with a mean of 2.2 kg. Twenty-six babies were preterm and mean gestational age of the study group was 34 weeks + 4 days. All the neonates admitted to this NICU had been transferred from outstations in Southern province. These admissions were for the management of various problems like prematurity, sub optimal birth weight, meconium aspiration, respiratory distress, infections etc. Probable risk factors which made the study participants more vulnerable for infections included very low birth weight, prematurity, cardiac anomalies, congenital diaphragmatic hernia, ventriculo-peritoneal shunt, abdominal surgery, prolonged mechanical ventilation, central lines for more than 48 h and intra-partum problems like meconium aspiration with birth asphyxia, obstructed-prolonged labour and premature rupture of membranes.

Twelve out of 50 admissions had clinically suspected EOS (240 per 1000 admissions) which was present on admission. LOS was noted in 43 occasions (860 per 1000 admissions) and 33 of this was considered to be acquired during 661 NICU patient days. The rest (n = 10) was identified on admission. Therefore, clinically suspected nosocomial infection rate was 50 per 1000 patient days. Culture proven rates for EOS and LOS were 2 and 15 episodes (40 and 300 per 1000 admissions), respectively. Culture proven nosocomial infection rate was 9 for 661 patient days (13.61 per 1000 patient days).

Gram negatives accounts for the majority of infections (70.5%) and septicaemia with no apparent primary focus was the commonest cause for positive blood culture (Table 1). In more than 90% of the occasions bacteraemia was due to coliforms and, extended spectrum beta lactamase (ESBL) producing *Klebsiella pneumoniae pneumoniae* was the commonest pathogen in blood cultures (72.7%) (Table 2) Almost all (90%) of the coliforms were resistant to beta lactam – beta lactamase inhibitors and third generation cephalosporin. Among aminoglycosides, gentamicin was the least sensitive while amikacin showed 7.7% resistance. On admission, the commonest surface colonizer was coagulase negative Staphylococcus (CoNS) (50%), followed by coliforms (40%) and methicillin resistant *Staphylococcus aureus* (MRSA) (10%). From rectal swabs, coliforms were isolated as the commonest (48.5%).

**Table 1:** Distribution of organisms in this study as per infections

Infection type	Number of cases	Organism (%)
Septicemia	6	Coliform (100%)
Shunt infection	1	Coliform (100%)
Nectrotizing enterocolitis	2	Coliform (100%)
Ventilator associated pneumonia	2	Coliform (100%)
Meningitis	3	Coliform (66.6%) *Streptococcus pneumoniae* (33.3%)
Congenital pneumonia	1	*Enterococcus* (100%)
Nosocomial pneumonia	2	*Acinetobacter* (50%) *CoNS* (50%)

**Table 2:** Distribution of pathogens in different types of clinical samples

Type of specimen	Pathogen	Number of episodes		
		Total	On admission	During NICU
Blood	ESBL producing coliforms (*Klebsiella pneumoniae pneumoniae*)	8	3	5
	Non-ESBL coliforms (*Proteus* spp.)	2	2	–
	*Strep. pneumoniae*	1	1	–
Respiratory secretions (*ET*, *PA*, *GA*)	ESBL producing coliform spp.	2	–	2
	*Enterococcus* spp.	1	1	–
	Coag. Neg. Staphylococcus	1	1	–
	*Acinetobacter* spp.	1	1	–
CSF (from EVD shunt)	ESBL producing coliforms	1	–	1

*ESBL* Extended spectrum beta lactamase; *ET* endo-tracheal; *PA* pharyngeal aspirate; *GA* gastric aspirate; *CSF* cerebrospinal fluid; *EVD* external ventricular drain

Relationship between clinical and surveillance cultures in LOS episodes acquired during the NICU stay is shown in Table 3. Sensitivity, specificity, positive predictive value and negative predictive value of surveillance swabs in predicting the organisms of late onset neonatal sepsis were calculated as are 77.8, 37.5, 31.8 and 81.8%, respectively. Five out of 7 (71. 4%) occasions where we isolated the pathogen of infection beforehand in surveillance cultures were from rectal swabs.

**Table 3:** Relationship between clinical cultures and surveillance cultures in LOS acquired during NICU stay (nosocomial infections)

LOS episodes acquired during NICU stay	Surveillance culture positive	Surveillance culture negative
Clinical culture positive (9)	7	2
Clinical culture negative (24)	15	9
Total (33)	22	11

Clinical cultures—cultures done on suspicion of infection

Surveillance cultures—cultures done on admission from anterior nares, umbilical swabs, respiratory secretions and rectal swabs and weekly during the NICU stay from respiratory secretions and rectal swabs

Surveillance culture positive—with regard to culture positive infection episodes, if we have isolated the same organism in a surveillance sample beforehand or with regard to culture negative infection episodes, if we have isolated a possible pathogenic organism as a colonizer in any surveillance sample

Surveillance culture negative—with regard to culture positive infection episodes, if we have not isolated the same organism or isolated a different organism in any of the surveillance samples beforehand. With regard to culture negative infection episodes, if we have not isolated any possible pathogenic organism as a colonizer they were also considered as screening negative occasions

### Discussion

Early prediction of pathogens of nosocomial sepsis is proven to be of value in starting effective first dose antibiotic therapy without delay and improving patient outcome [6, 7]. Nosocomial pathogens are known to be acquired in different ways; cross infection from other infected patients, colonized healthcare workers, contaminated equipment and the endogenous flora of the patient him/herself [11–13]. Therefore surveillance cultures were used in this study to identify potential pathogens in endogenous flora.

The value of routine surveillance of surface colonizers in neonates is a controversial issue. According to Jolley some studies show surface swabs are inefficient and not cost effective for guiding empirical therapy of neonatal sepsis while some have shown value with surveillance ET cultures in perinatal pneumonia [14]. In 77.8% of the nosocomial infection episodes, we can predict the causative pathogen using the isolates in surveillance samples, which is much higher than the findings of De Jong, where they could predict the potential pathogen in 41% of the infections by surveillance cultures [15]. Most useful surveillance culture type was rectal swabs, which yielded the same organism as in clinical sample in 71.4% occasions. This is a useful finding, especially in order to prevent use of inappropriate antibiotics in empirical therapy. On the other hand, collecting surface swabs is non-invasive and easy to perform on neonates.

Since high prevalence of antimicrobial resistance in the unit can affect the effective empirical antibiotic therapy, we analyzed all the clinical cultures and found that there is a high discrepancy between clinically suspected infection rate and the culture proven rate. This could be due to several reasons like low threshold for clinical suspicion of sepsis in neonates, commencing antibiotic therapy prior to collection of cultures and practical difficulties in repeated and adequate sample collection for cultures from neonates.

Overall nosocomial infection rate of culture proven sepsis, 13.61 episodes per 1000 patient days seems to be higher than in studies from developed countries. Neonatal nosocomial infection rates across the Australian and New Zealand network 2009, had found an overall rate of 5.02 episodes of infections per 1000 patient days for infants of less than 1000 g birth weight [16]. However, blood stream infection rate of 7.56 per 1000 patient days is compatible with the findings in Gastmeier et al. which have recorded a rate of 6.4 per 1000 days in a less than 1000 g birth cohort from Berlin. [17]. Gram negative organisms predominated as pathogens, which is similar to what was reported from neonatal units in other developing countries [18, 19]. Two clusters of *Klebsiella pneumoniae pneumoniae* bacteraemias occurred during this period also has accounted for the high proportion of Gram negatives. Group B streptococcus was not isolated in any of the samples, and can be due to effective ante-partum screening and intra-partum antibiotics. Studies have shown a shift from Gram positives to Gram negatives over the period of time. Gladstone et al. have described group A streptococci and *S. aureus* as the predominating pathogens in the pre-antibiotic era, while in 1940s and 1950s Gram negative organisms, particularly *Escherichia coli* has become the most common pathogen [20, 21].

Considering the ABST patterns of coliforms in culture proven LOS we recommend carbapenems and aminoglycosides as the most effective empirical antibiotic therapy. This is emphasized by the fact that rectal swabs being the most useful surveillance cultures, which isolated ESBL producing coliforms. Different findings have been obtained from another study recommending combined vancomycin and amikacin for empirical therapy [22].

### Conclusions

Overall, surveillance cultures have a good sensitivity in prediction of pathogens of LOS in neonates. Nasal swabs, respiratory secretions and umbilical swabs are of limited value as screening samples. However, routine surveillance with rectal swabs is useful in neonates to predict the likely pathogens of LOS and guiding the empirical antibiotic therapy of nosocomial LOS.

## Limitations

Due to limited time factor available for this study and considering the number of admissions to this unit we had to limit the sample size to 50. Anaerobic cultures could not be done due to limited resources.

## Abbreviations

ABST: antibiotic sensitivity test
CoNS: coagulase negative *Staphylococcus*
EOS: early onset sepsis
ESBL: extended spectrum beta lactamase
ET: endotracheal
EVD: external ventricular drain
GA: gastric aspirate
LOS: late onset sepsis
MRSA: methicillin resistant *Staphylococcus aureus*
NEC: necrotizing entero-colitis
NICU: Neonatal Intensive Care Unit
PA: pharyngeal aspirate
THK: Teaching Hospital Karapitiya
VAP: ventilator associated pneumonia
